# Do Microplastics Enter Our Food Chain Via Root Vegetables? A Raman Based Spectroscopic Study on *Raphanus sativus*

**DOI:** 10.3390/ma14092329

**Published:** 2021-04-30

**Authors:** Leda-Eleni Tympa, Klytaimnistra Katsara, Panagiotis N. Moschou, George Kenanakis, Vassilis M. Papadakis

**Affiliations:** 1 Institute of Molecular Biology and Biotechnology, Foundation for Research and Technology–Hellas, N. Plastira 100, GR-70013 Heraklion, Greece; leda_tympa@imbb.forth.gr (L.-E.T.); klytaimnistra_katsara@imbb.forth.gr (K.K.); panagiotis.moschou@imbb.forth.gr (P.N.M.); 2 Department of Biology, University of Crete, N. Plastira 100, GR-70013 Heraklion, Greece; 3 Department of Plant Biology, Uppsala BioCenter, Linnean Center for Plant Biology, Swedish University of Agricultural Sciences, P.O. Box 7080, S-75007 Uppsala, Sweden; 4 Institute of Electronic Structure and Laser, Foundation for Research and Technology–Hellas, N. Plastira 100, GR-70013 Heraklion, Greece; gkenanak@iesl.forth.gr

**Keywords:** label-free, microscopy, detection, plastic, pollution, environment, edible, crop plants, ABS

## Abstract

The outburst of plastic pollution in terrestrial ecosystems poses a potential threat to agriculture and food safety. Studies have already provided evidence for the uptake of plastic microparticles by several plant species, accompanied by numerous developmental effects, using fluorescence labelling techniques. Here, we introduce the implementation of confocal Raman spectroscopy, a label-free method, for the effective detection of microplastics (MPs) accumulation in the roots of a common edible root vegetable plant, *Raphanus sativus*, after treatment with acrylonitrile butadiene styrene (ABS) powder. We also demonstrate the concomitant occurrence of phenotypic defects in the polymer-treated plants. We anticipate that this work can provide new insights not only into the extent of the impact this widespread phenomenon has on crop plants but also on the methodological requirements to address it.

## 1. Introduction

Due to its presence in nearly all parts of the biosphere, the potential impact of plastic pollution is not easy to define, thus creating risks for both humans and the environment [1]. Principally, polymers can be either artificially manufactured (synthetic plastics) or pre-existing (naturally occurring plastics). Both synthetic plastics (e.g., LDPE) and their naturally occurring homologues (e.g., wool fibers) include non-biodegradable and biodegradable polymers. Nevertheless, the environmentally friendly process of plastic biodegradation, depends highly on the environmental conditions, often resulting in incomplete mineralization of such polymers [2].

Terrestrial plants are directly exposed to plastic pollution, deriving from a plethora of sources, such as the application of sewage sludge and organic fertilizers, agricultural plastic film or the atmospheric deposition of airborne particles. Plastic debris, characterized by a variety of sizes, can be subsequently degraded into MPs (less than 5 mm by natural weathering processes [3]. Furthermore, although the effects of MPs contamination on human health remain understudied, they may lead to cell damage and/or trigger inflammatory and immune reactions [4].

The migration of MPs to edible plant tissues has been demonstrated [5,6], while the presence of MPs in the soil-plant system can affect various developmental stages negatively or positively, depending on the plant species [7,8,9]. Qi et al. (2018) investigated the effects of plastic mulch film MPs on wheat using low-density polyethylene (LDPE) and biodegradable plastic. The results revealed negative impacts on both subterranean and external organs of wheat, regarding both vegetative and reproductive development [9]. A recent study from Bosker et al. (2020), showed that MPs can accumulate on pores in seed capsule and delay germination and root growth of *Lepidium sativum* terrestrial vascular plants [7]. On the contrary, studies on *Arabidopsis* and wheat showed that no MPs accumulated in plant tissues beyond root cap [10], while Zhang et al. (2015) reported that high concentrations of LDPE particles can improve soil fertility [11].

Interestingly, the proposed mechanism for the uptake of MPs in crop plants is the “crack-entry” pathway, via the apoplastic transport system (through-cell wall). Root openings that develop as a consequence of ageing, and damage by below-ground herbivores and mechanical injury, might also provide entry sites for MPs. Once in the central cylinder, particles are systematically self-assembling and being transferred from the roots to the stems and leaves via the vascular system, following the transpiration stream [12].

The implementation of traditional detection methods (i.e., transmission electron microscopy, TEM, and scanning electron microscopy, SEM) [5] and fluorescence labeling techniques and confocal laser scanning microscopy (CLSM) [6] have been so far very helpful for the monitoring of MPs accumulation and dispersion in plant tissues. However, the required fixation and sample preparation steps are invasive and can be laborious and time-consuming.

Vibrational spectroscopic techniques can provide highly detailed chemical and compositional information of label-free samples, being applied in situ, with minimum preparation required. Raman spectroscopy is such a label-free technique, used to provide structural fingerprints for material identification, based upon the interaction of light with the chemical bonds present within a molecule. The application of Raman spectroscopy in biology is extensive due to its multiple advantages compared to other standard spectroscopic techniques (e.g., FT-IR). Specifically, Raman spectroscopy does not interfere with water molecules and the spot size of the measurement is relatively small. Furthermore, recent advances in Raman spectroscopy can significantly enhance the signal to noise ratio by the use of nanoparticles, enabling the detection of specific biomolecules [13,14,15]. Lastly, another significant advantage of Raman spectrometers is that currently, they become also available in a portable form that allows operation even in the field.

In our work, we investigated whether a crop plant species with subterranean edible parts, *Raphanus sativus* (common radish), accumulates plastic debris when growing on a substrate incidentally or repeatedly exposed to plastic pollution in a label-free fashion, using confocal Raman spectroscopy. Particularly, to test our hypothesis, the MPs used in this work had a diameter smaller than 100 μm, to enable their migration into the plant roots. Our results can contribute to a better understanding of both the extent and the mechanisms through which MPs enter the roots and affect plant development. Moreover, this work can assist in enriching the methodological toolbox of a putative MPs field-level detection in crop plants, with further interest in food safety and security, and human health.

## 2. Materials and Methods

### 2.1. FE-SEM Characterization of ABS MPs Powder

**Figure 1:**
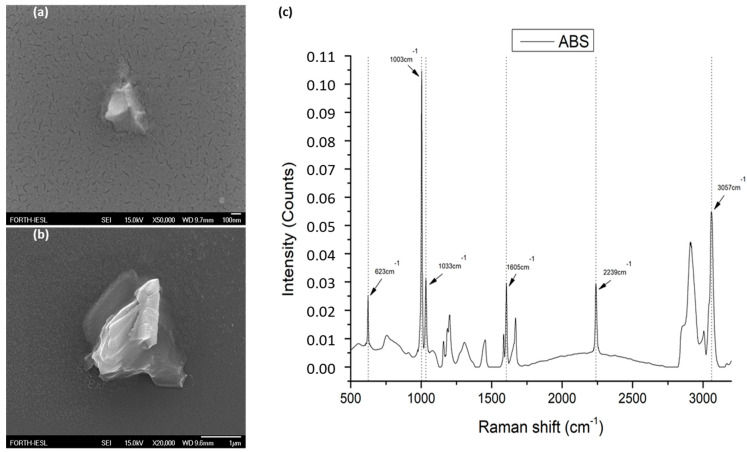
ABS powder characterization through FE-SEM indicative images from MPs: (**a**) one of the smallest MPs with approximately 0.3 μm diameter; (**b**) a MP with around 2 μm diameter; (**c**) the characteristic Raman spectrum measured indicating the identified Raman peaks present in the microplastics in radish roots.

Acrylonitrile-butadiene-styrene (ABS) was provided by (INEOS Styrolution, Frankfurt, Germany). The ABS was industrial grade, delivered in the form of a fine powder of variable sizes (approximately 1–300 μm), under the name Terluran Hi-10. The powder used in the presented work was firstly characterized by field emission scanning electron microscopy (FE-SEM, JSM-7000F, JEOL Ltd., Tokyo, Japan). This enabled us to confirm the physical dimensions of the microparticles that were added to the radish plants treatment. The physical dimensions of the ABS microparticles varied between 0.3 and 100 μm. In a parallel step, we performed Raman spectroscopic characterization to confirm that the particle dust was indeed ABS (Figure 1).

### 2.2. Experimental Methodology and Plant Material Preparation

**Figure 2:**
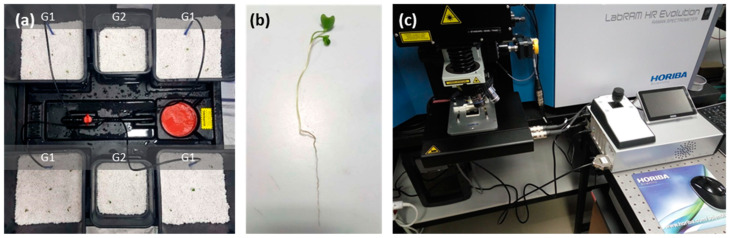
Experimental setup for the spectroscopic detection of ABS in radish roots: (**a**) Hydroponic cultivation of radish plants. (**b**) Treated sample before sectioning and microscopic observation. (**c**) LabRAM HR Raman Spectrometer confocal Raman microscope.

*Raphanus sativus* plants (variety “White Icicle”) were hydroponically grown on perlite with the use of an active irrigation system Wilma (Atami, Valencia, Spain), in a semicontrolled chamber PROBOX (Garden HighPro, Barcelona, Spain). The seeds were vernalized for four days at 18 °C and in turn, grown at 25 °C, 8 h light 23 °C/16 h dark 20 °C cycles, 12,600 lumen light intensity, 75–85% relative humidity), for a maximum of three weeks. The irrigation solution was supplied with a basic grow nutrient (powder feeding grow) and switched to a root nutrient (Canna Rhizotonic) by the beginning of the second week. ABS powder was applied to one group of plants (G1), in contrast to the second group of plants, which were used as a negative control (G2, Figure 2a). Two experiments (E1 and E2) were conducted, differing in the methodology used to provide ABS powder to the plants. In E1, ABS was initially mixed with the perlite substrate (10% *w*/*w*), while in E2 ABS was applied to the plants by periodic irrigation (10% *w*/*v*). Transverse sections of thoroughly washed fresh roots were handmade with a medical scalpel and observed under a confocal microscope for the spectroscopic detection of ABS (Figure 2b,c).

### 2.3. Measurement Instrumentation and Settings

Raman measurements were made by a modified LabRAM HR Raman Spectrometer (HORIBA Scientific, Lille, France). The Raman excitation laser line used had a central wavelength at 532 nm and a maximum laser output power of 90 mW. The objective lenses used were two: one 10× for imaging and second 50× for imaging and Raman measurements, with a numerical aperture of 0.5 and a working distance of 10.6 mm, both LMPlanFL N (Olympus, Tokyo, Japan). The resulting maximum laser power on the sample under the aforementioned setup was 5 mW. The laser spot spatial diameter was approximately 1.7 μm, with an axial length of about 2 μm. A grating of 600 groves was used that resulted in a Raman spectral resolution of around 2 cm^−1^.

The Raman signal detector was the Syncerity CCD Deep Cooled Camera (HORIBA), operating at −50 °C. A temperature-controlled stage (PE120-XY, Linkam) was coupled with the microscope stage that kept the sample temperature constant of T = 18 °C. Spectral calibration was performed with a SiO_2_ reference sample, presenting a single peak at 520.7 cm^−1^. The microscope was set to acquire Raman signal in the spectral range between 300 and 3300 cm^−1^ resulting in 2 spectral acquisition windows. Acquisition time was set to 1 s, with a spectral accumulation of 3, resulting in a total acquisition time of around 7 s for every point. Raw Raman spectral data underwent the following processing procedure: (a) cosmic rays were removed by an internal function of LabSpec software LabSpec LS6 (HORIBA Scientific, Lille, France); (b) background signal was subtracted from the raw spectral data using a polynomial function; and (c) processed data were analyzed by a custom laboratory software tool, used to identify the Raman peaks.

## 3. Results

### 3.1. Phenotypic Observations

**Figure 3:**
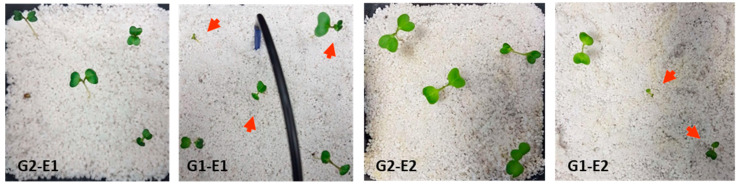
Radish seedlings from different groups (G) two weeks post sowing, in the two experimental setups (E). Phenotypic aberrations are indicated by red arrows.

In both experiments, plants that were treated for two weeks with ABS powder were characterized by higher developmental variability and cotyledon shape and size (quantified by the maximum length of the line perpendicular to the cotyledon central nerve), in contrast to the untreated plants, with more robust development appearing well-formed (Figure 3, Table 1).

### 3.2. Detection of MPs in Radish Root Sections

**Figure 4:**
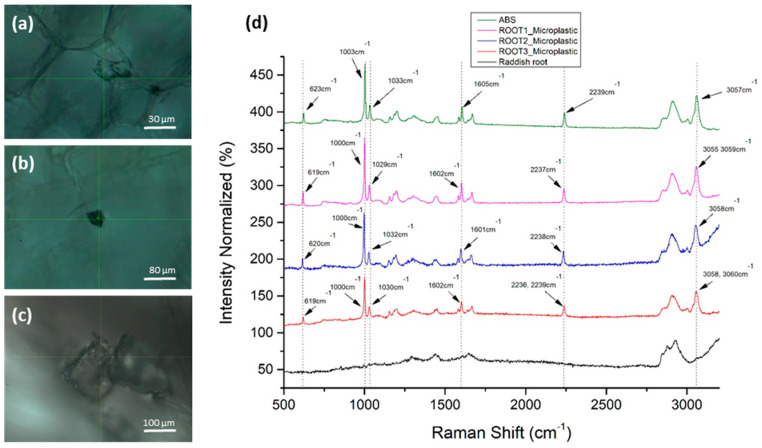
Detection of ABS MPs in radish roots: (**a**–**c**) Confocal MPs images in root sections (brightfield); (**d**) Raman spectra of the experimentally detected MPs (purple, blue and red), radish root background (black) and ABS powder (green line).

Radish root sections were scanned at three different developmental stages for the presence of MPs. Specifically, confocal Raman scans were performed for a one-week-old (a) and a two-week-old (b) seedling in the first experimental setup, and a three (c) week-old seedling in the second experimental setup. In all cases, the confocal localization of MPs inside the root tissue was followed by the detection of the ABS reference spectrum, obtained from ABS powder (Figure 4). The identified microplastics were significantly larger than the focal point dimensions of the laser (1.7 μm spatial, 2.0 μm axial).

Ιn Figure 4d, we observed that the six main Raman peaks of the ABS polymer were identified in the microparticle images of Figure 4a–c in the radish roots, which confirms their identity as ABS aggregates. Raman peak wavenumbers were within the acceptable technical deviations (±4 cm^–1^). The microparticle’s total Raman fingerprint corresponds to pure ABS polymer. The Raman peaks of ABS and MPs with their assignments are presented in Table 2.

### 3.3. Observations of MPs Spectroscopic Alterations in Later Developmental Stages

**Figure 5:**
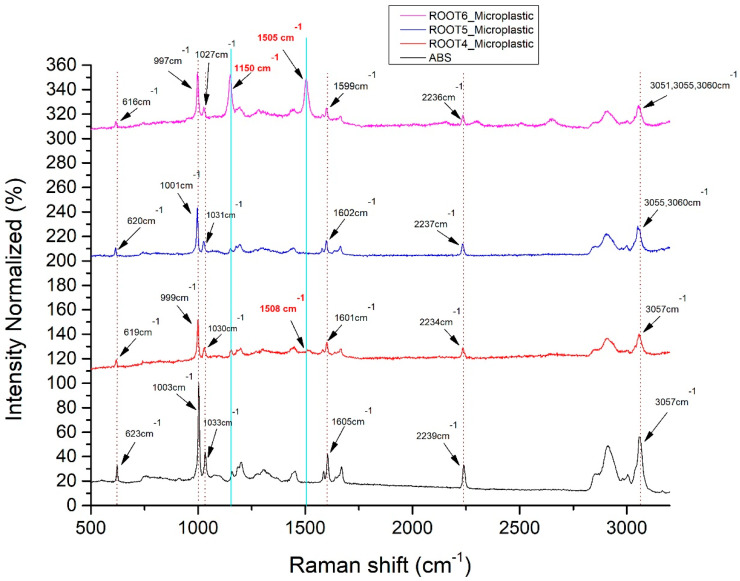
Detection of different spectra between ABS MPs in radish roots (purple, blue and red) and ABS powder (black). Raman fluctuating peaks are indicated with bold red.

As shown in Figure 5, the six main Raman peaks of the ABS polymer were identified in the microparticles at the later developmental stage of radish roots. This also confirms their identity as ABS aggregates (see also Figure 4d). The Raman peaks of microparticles were again within the acceptable measurement technical deviations (±4 cm^−1^). In contrast, the Raman signal from the microparticles did not correspond purely to the ABS polymer fingerprint. Some extra Raman peaks were randomly appearing, due to the possible development of some microbial load within the time that MPs remained inside the plant. The Raman peaks of ABS and MPs with their assignments are presented in Table 3.

Further investigation of ABS MPs identified in the roots of eight-week-old radish in the second experimental setup revealed spectral fingerprints that deviate from the ABS reference spectrum (Figure 5, Table 4). Interestingly, an intensity increase regarding the characteristic peak at 1150 cm^−1^ is observed. Additionally, the appearance of a fluctuating peak at 1508 cm^−1^ and 1505 cm^−1^, corresponding to N-H bending, cytosine or acetyl coenzyme A could be potentially indicating the development of biological activity or deposition of material around the respective scanned particles.

### 3.4. Sequential Confocal Localization of an Identified MP within the Root Tissue

**Figure 6:**
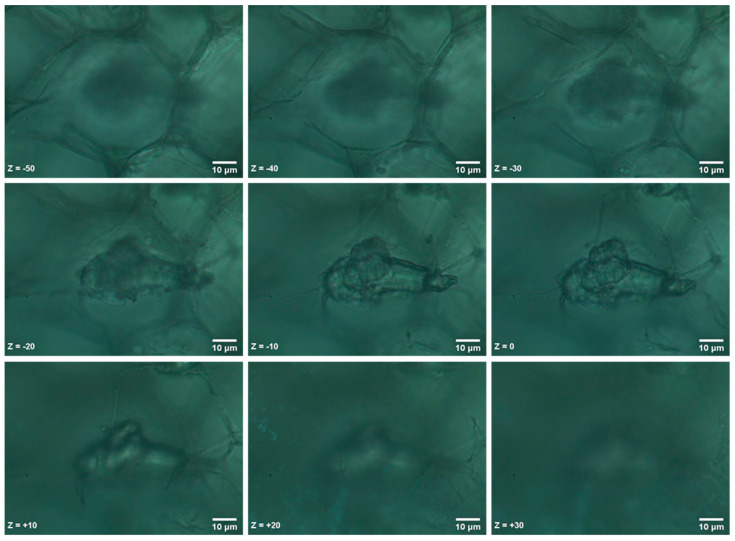
Confocal localization of an ABS MP at different Z positions within the root section.

The identified MPs were localized within the radish root tissue and not randomly precipitated on the surface of the section. In the following example, we acquired multiple sequential images in a 10 μm interval on the Z axes. Initially, we focused at the geometrical center of the MP, which we set as the zero position (Z = 0), and acquired images starting at 50 μm above the MP surface (Z = −50) and focusing down to 30 μm under the MP section (Ζ = +30). The sequential images are shown in Figure 6 and were also used to construct a Z-Stack animation (Z-Stack.GIF), which is provided in the Supplementary data of this paper.

## 4. Discussion

Our findings verify that plastic microparticles and nanoparticles can migrate and accumulate in the tissues of edible crop plant species [5]. Furthermore, MPs can localize within the root tissues as shown through the sequential confocal image sections. Additionally, we observed inconsistencies in the cotyledon size and shape of plants treated with MPs. This result could indicate the development of a discontinuity in the vascular system due to the plastic debris aggregation, which would lead to insufficient nutrient transfer. The presumed mechanism through which ABS powder can flow into the apoplastic pathway is the already proposed one by Li et al. (2020) [6]. Specifically, plastic particles up to 2 μm in size with a small degree of mechanical flexibility can compress into the apoplastic space of plant root cells, or into small cracks where lateral roots emerge from, subsequently entering the xylem vessels, while the possibility that larger particles can also be uptaken following this accumulation pattern is also discussed.

ABS is slightly hydrophobic due to abundant methylene and phenyl groups, [21] creating an 81.0 contact angle with water. Despite this, ABS due to the presence of residual emulsifier and the polarity of the nitrile side groups shows an absorption amount of up to 1.5% of water upon storage in aqueous media such as our plant cellular environment [22]. Depending on the percentage of trapped air, and the shape of the ABS MPs, they have corresponding self-organization and hydrophilicity-hydrophobicity into the plant. Upon longer exposure, ABS can dissolve in water reducing the viscosity, which will result in self-organization and aggregations of MPs [23]. In a relevant publication [24], it was confirmed that ABS can behave both as a super-hydrophilic and super-hydrophobic polymer. The MPs used in this experiment had similar surface roughness, which could be an explanation for the aggregates formed in the plant tissue.

Noticeably, spectral fluctuations were also observed, corresponding to accumulated MPs scanned within the tissues of radishes beyond the fifth week of development. Intensity fluctuations of the Raman peak at 1150 cm^−1^ could be reflecting changes in carotenoids during development in the radish root surrounding tissue [25]. Moreover, a peak appearing solely in two of the scanned radish roots at 1505 cm^−1^ and 1508 cm^−1^ and assigned to N-H bending, cytosine or acetyl coenzyme A, could be associated with biodeterioration procedures taking place upon the respective ABS particle. MPs may lead to increased endophytic microbial content or changes in quantitative or qualitative tissue parameters. Such events could derive from fungal or bacterial colonization. Specifically, certain microorganisms are capable of utilizing plastic as a substrate for their development, a process that has already been described for other types of plastic. Furthermore, MP aggregation may lead to changes in the biophysical properties of tissues by exerting pressure on biomaterials, such as the cell wall [26].

Herein detection of ABS was done by Raman spectroscopy, a label-free technique with minimum sample preparation requirements. Regarding the respective technical restrictions within the framework of this study, MPs monitoring in the plant tissue and further investigation of the ABS accumulation mechanism were not conducted, since they were out of the scope of this study. Based on the concept of this work, (a) spectroscopic characterization could be regarded as a promising tool for field-level analysis of agriculturally important crops exposed to MPs pollution, (b) future studies will further elucidate the long term effects of MPs uptake on the development of different plant species, under actual field conditions and (c) new insights will be provided into this widespread phenomenon, with a particular interest in food safety and security issues, and ultimately human health.

## 5. Conclusions

Our study establishes that radish plants can accumulate exogenously provided MPs (ABS powder) within their roots. Specifically, we have exhibited that the detection of MPs in plant tissues can be effectively achieved by confocal Raman spectroscopy. Interestingly, radish plants treated by ABS MPs have appeared phenotypically diversified from untreated plants, while accumulated ABS MPs have displayed spectral fluctuations after the 8th week of development. Overall, it is evident that Confocal Raman Spectroscopy could be regarded as a promising tool in various field-level studies regarding MPs pollution.

## Figures and Tables

**Table 1:** Cotyledon width variability within the groups (G) of radish seedlings, in the two experimental setups (E). Cotyledon width values are linearly correlated with the color saturation of the heatmap to enhance visualization of width variability.

Variability of Cotyledon Size	Plant Group			
Width (Pixels)	G2-1	G2-1	G1-1	G1-2
1	129.560	113.799	197.428	60.828
2	120.340	98.984	63.334	36.962
3	97.798	109.863	14.981	95.789
4	101.546	104.546	15.114	85.255
5	83.613	108.912	83.205	21.427
6	77.517	101.544	71.653	19.067

**Table 2:** Raman peaks of ABS and MPs with their assignments.

Raman Peaks (cm^−1^) of ABS	Raman Peaks (cm^−1^) of Root1 mp	Raman Peaks (cm^−1^) of Root2 mp	Raman Peaks (cm^−1^) of Root3 mp	Assignments Referred to ABS [16]
623	619	620	619	621 cm^−1^ → d (ring) of benzene
1003	1000	1000	1000	1002 cm^−1^ → Benzene ring breathing
1033	1029	1032	1030	1032 cm^−1^ → δ (C–H) in plane of Benzene
1605	1602	1601	1602	1603 cm^−1^ → vs(C–C) of benzene ring
2239	2237	2238	2236, 2239	2239 cm^−1^ → v (C≡N)
3057	3055, 3059	3058	3058, 3060	3060 cm^−1^ → v (=C–H) of benzene ring

**Table 3:** Raman peaks of ABS and MPs with their assignments.

Raman Peaks (cm^−1^) of ABS	Raman Peaks (cm^−1^) of Root4 mp	Raman Peaks (cm^−1^) of Root5 mp	Raman Peaks (cm^−1^) of Root6 mp	Assignments Referred to ABS [16]
623	619	620	616	621 cm^−1^ → d (ring) of benzene
1003	999	1001	997	1002 cm^−1^ → Benzene ring breathing
1033	1030	1031	1027	1032 cm^−1^ → δ (C–H) in plane of Benzene
1605	1601	1602	1599	1603 cm^−1^ → vs(C–C) of benzene ring
2239	2234	2237	2236	2239 cm^−1^ → v (C≡N)
3057	3057	3055, 3060	3051, 3055, 3060	3060 cm^−1^ → v (=C–H) of benzene ring

**Table 4:** Fluctuating Raman peaks (cm^−1^) and their assignments as detected in the later developmental stages of radish root sections.

ABS	Root1 mp	Root2 mp	Root3 mp	Root4 mp	Root5 mp	Root6 mp	Assignments
1157	1155	1150	1153	1151, 1155	1151	1150 strong	1156-1157 cm^−1^ → δ(C–H) out-of-plane of benzene ring in ABS [16] 1157 strong cm^−1^ → CC, CO stretching asymmetric, (crystalline) in cellulose [17] 1151 cm^−1^ → shoulder in cellulose [18] 1150 cm^−1^ → Glycogen, Carotenoid [19] 1152 cm^−1^ → ν(C–N), proteins (protein assignment), ν(C–C) carotenoids, Carotenoid peaks due to C–C and conjugated C=C band stretch [19] 1153 cm^−1^ → Carbohydrates peak for solutions [19] 1154 cm^−1^ → -Carotenes [19] 1155-1157 cm^−1^ → Carotenoids [19] 1155 cm^−1^ → C–C (and C–N) stretching of proteins (also carotenoids), Glycogen, ν (C–C)- Diagnostic for the presence of a carotenoid structure, most likely a cellular pigment [19] 1156 cm^−1^ → C–C, C–N stretching (protein) [19] 1157 cm^−1^ → In-plane vibrations of the conjugated =C–C=, β-carotene accumulation (C=C stretch mode) [19]
-	-	-	-	1508 (weak)	-	1505 (strong)	1506 cm^−1^ → N–H bending [19] 1506 and 1508 cm^−1^ → Cytosine [19] 1508 cm^−1^ → Acetyl coenzyme A [20]

## Data Availability

All data reported here can be made available upon request.

## Supplementary Materials

The following are available online at https://www.mdpi.com/article/10.3390/ma14092329/s1.
