# Supplementary figures and images for: Do Microplastics Enter Our Food Chain Via Root Vegetables? A Raman Based Spectroscopic Study on Raphanus sativus
Source: Materials (Basel). 2021 Apr 30;14(9):2329. doi: 10.3390/ma14092329 (PMC8124748; doi:10.3390/ma14092329)

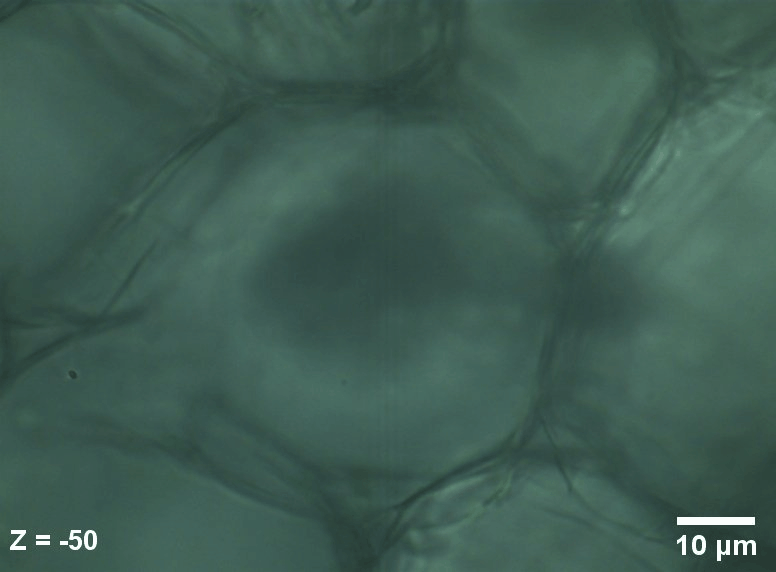

Supplement: Supplementary file 1 [file materials-14-02329-s001.zip › supplementary.gif]
